# Pandemic detention: life with COVID-19 behind bars in Maryland

**DOI:** 10.3389/fpubh.2023.1217857

**Published:** 2023-07-20

**Authors:** Joyell Arscott, Brandon Doan, Lauren Dayton, Gabriel B. Eber, Carolyn B. Sufrin, Chris Beyrer, Leonard Rubenstein

**Affiliations:** ^1^Department of Epidemiology and Prevention Science, Johns Hopkins Bloomberg School of Public Health, Baltimore, MD, United States; ^2^Department of Health, Behavior and Society, Johns Hopkins Bloomberg School of Public Health, Baltimore, MD, United States; ^3^Center for Public Health and Human Rights, Department of Epidemiology, Johns Hopkins Bloomberg School of Public Health, Baltimore, MD, United States; ^4^Department of Gynecology and Obstetrics, Johns Hopkins School of Medicine, Baltimore, MD, United States; ^5^Duke Global Health Institute, Duke University, Durham, NC, United States

**Keywords:** COVID-19, Maryland, pandemic (COVID-19), incarceration, re-entry, release process, detention centers

## Abstract

**Background:**

People incarcerated during the COVID-19 pandemic face higher vulnerability to infection due to structural and social factors in carceral settings. Additionally, due to the higher prevalence of chronic health conditions among carceral populations, they are also at risk for more severe COVID-19 disease. This study was designed to explore the experiences of people incarcerated in prisons and jails in Maryland during the height of the pandemic.

**Methods:**

We conducted semi-structured phone interviews between January 2021 and April 2022 with ten individuals incarcerated in Maryland carceral facilities during the height of the U.S. COVID-19 pandemic and were subsequently released from prison or jail. We transcribed the interviews, coded them, and engaged in content analysis, an inductive analytical approach to developing themes and meaning from qualitative data.

**Results:**

Four themes emerged from participants’ descriptions of their experiences: (1) distress from fear, vulnerability, and lack of knowledge about COVID-19 and how to protect themselves, (2) shortcomings of prison and jail administrators and other personnel through lack of transparency and arbitrary and punitive enforcement of COVID-19 protocols, (3) lack of access to programming and communication with others, and (4) absence of preparation for release and access to usual re-entry services.

**Conclusion:**

Participants responded that the prison and jails’ response during the COVID-19 pandemic was ill-prepared, inconsistent, and without appropriate measures to mitigate restrictions on liberty and prepare them for release. The lack of information sharing amplified their sense of fear and vulnerability unique to their incarceration status. Study findings have several institutional implications, such as requiring carceral facilities to establish public health preparedness procedures and making plans publicly available.

## Introduction

The COVID-19 pandemic posed a particularly severe threat to the health of people incarcerated in prisons, jails, and other detention facilities. These facilities are subject to high population densities, confined in tight spaces with constrained access to hygiene and cleaning supplies, and the persistent risk of transmission from the daily flux of workers entering and leaving facilities (1, 2). At the same time, there is a high prevalence of certain chronic diseases among incarcerated people in the United States, making them especially vulnerable to severe cases of COVID-19. For instance, in a nationwide survey, more than half of state prisoners reported ever having a chronic condition, and 43% reported a current chronic condition. These included, among others, chronic lung disease or asthma, heart conditions and cardiovascular diseases, obesity, and diabetes (3). These conditions, along with stress, anxiety, and other mental health factors, place individuals who are incarcerated at increased risk for severe illness (4).

Exposure to and mortality from COVID-19 in carceral settings was especially high early in the pandemic: between April and June 2020, people in prison were five and a half times more likely to be infected with COVID-19 and three times more likely to die from COVID-19 compared to the general population, after adjusting for age and sex (5). During this time, 8 of 10 of the largest COVID-19 outbreaks in the U.S. occurred in prisons and jails (6). State and federal carceral facilities reported that, between March 2020 and February 2021, 8.2% (396,300) of incarcerated persons tested, tested positive for COVID-19, with 2,500 COVID-19-related deaths (7).

The high risk of COVID-19 for carceral populations is not only critical to address for the health of people who are incarcerated but also affects the health of surrounding communities. COVID-19 infection spreads to and from incarcerated persons and surrounding communities through now well-understood pathways. The daily rotation of prison and jail staff and detainees’ intake, transfer, and release are likely sources of viral transmission between carceral settings and the general community. Prison staff have COVID-19 infection rates higher than the general population, but similar to COVID-19 rates of the incarcerated population (8, 9). An April 2020 study reported that 16% of all COVID cases in the state of Illinois could be traced back to jail-community cycling at the Cook County jail (10). Additional studies also found that movement between local prisons and jails and surrounding communities contributed to rising COVID-19 community infections (11–13).

Given the evidence that incarcerated persons are at increased risk for COVID-19 transmission, the CDC released guidelines for COVID-19 control in detention facilities, but not until several months after the onset of the pandemic. Many of these guidelines were not well implemented and were slow to be updated with new research and practices (14–18). Little is known about how people incarcerated in the U.S. experienced prison and jail administrators’ responses to COVID-19 control measures.

One COVID-19 mitigation strategy was to decrease the number of people behind bars by limiting pre-trial detention and facilitating early releases (19). At the same time, however, the COVID-19 pandemic also considerably disrupted re-entry planning. However, little is known about the first-hand re-entry experiences of those leaving prison or jail during the pandemic.

Evidence of the unique interconnected health risks between custody officers and incarcerated people is situated within complex and often adversarial relationships (20). Nevertheless, there is still much to learn, particularly from the perspectives of those who directly experienced being incarcerated in the U.S. setting during the intense early phase of the COVID-19 pandemic, but only a handful of researchers have assessed the perceptions of people directly impacted by these policies (21, 22). To address this gap, we conducted a qualitative study to understand the lived experiences of formerly incarcerated individuals who were incarcerated and released during the COVID-19 pandemic in the state of Maryland. Their perspectives on the early pandemic response offer unique insights for clinicians, researchers, public health professionals, and administrators to improve response, lower mortality, and best practices for incarcerated populations during a future pandemic.

## Methods

### Study design

A phenomenological approach was used to explore the lived experiences of individuals incarcerated and released during COVID-19.

### Study setting

The study was conducted among people incarcerated in Maryland prison, jails, and federal prisons. The state has a total of 24 state prisons and 30 jails. By the end of 2020, federal and state prisons in Maryland housed a total of 15,623 people (*n* = 15,623 men, *n* = 518 women), with 3,047 newly admitted that year (23). In the same year, 5,933 incarcerated individuals were released. By Spring 2021, the total number of people incarcerated in Maryland was reduced to 14,963 people (24). 70.9% of Maryland’s incarcerated population is Black, 22.6% is White, and 4.7% is Hispanic (23).

### Study sample

We recruited participants residing in Maryland who had been incarcerated in a Maryland or federal detention prison or jail and released during the COVID-19 pandemic after March 1, 2020, when the COVID-19 pandemic began in earnest. Recruitment activities lasted 16 months, beginning in January 2021 and ending in April 2022. We aimed to enroll formerly incarnated participants to understand their incarceration and release experiences during the COVID-19 pandemic. Participants were motivated to share their experiences during COVID-19 to raise awareness and hope to impact change. Eligibility criteria included age over 18 years and the ability to speak English. Persons were deemed eligible for screening once released from prison or jail with no time constraints. Recruitment targeted an equal representation of both men and women participants to compensate for an incarcerated population that is disproportionately men. All recruitment activities occurred in collaboration with four regional partner community organizations serving persons currently or previously incarcerated, distributing flyers and study announcements to their clients. We also utilized snowball sampling and online Facebook advertisements targeting adults in the Maryland area who may know someone who was incarnated during COVID-19.

### Protection of human subjects

The study protocol and human subjects’ protection approval was obtained from the Johns Hopkins Bloomberg School of Public Health Institutional Review Board.

### Data collection

A semi-structured interview guide was developed to conduct the study. The interview guide was designed around the study’s objective to understand the impact of COVID-19 mitigation on incarcerated access to information about the disease, prevention measures such as personal protective equipment (PPE), and resources such as access to legal counsel, medical and mental health care related directly and indirectly to COVID-19, and an efficient and fair process for release from custody. We designed the interview guide to explore these key aspects of incarceration and health care that early reports identified as influenced by the pandemic (19). Community partners provided input on the interview guide, and the research team made revisions accordingly. All individual interviews were conducted via telephone between January 2021 and April 2022. Interviews were conducted via phone, scheduled at a time that was convenient for participants. The interviews lasted an average of 30 min but ranged from 10 min to 58 min. Each participant received a $25 gift card at the end of the interview as an honorarium for participation.

The guide’s open-ended questions asked participants to provide their narrative accounts of their experiences through such questions as “Did you experience any changes in how you could access health care services in jail/prison because of COVID;” “What obstacles did you face, if any, in getting released;” and “What did custody officers do to prevent the spread of COVID-19?” The guide also included closed-ended questions. Closed-ended questions included demographics (e.g., “How old are you?” and “How would you describe your race/ethnicity?”). Additionally, closed-ended questions probed on infection control measures (e.g., “Did -custody officer wear masks?”), access to services while in custody (e.g., “Were you tested for COVID-19 prior to release?”), and re-entry challenges (e.g., “Have you tried to access behavioral or mental health services?”). These questions were used to describe the characteristics of the study population (Appendix A).

All interviewers had extensive experience conducting and analyzing qualitative interviews. The interviewers received training on the content of the interview guide and how to use google voice, the software used to conduct and record the interviews. Interviewers were also trained to ask participants if they were in a quiet and private location and to conduct informed consent. Each interviewer conducted a test interview, and other study team members listened to the recording and provided feedback during the interviewer training, which occurred before conducting the interviews and was ongoing over the study period. During the study period, interviewers met weekly to discuss interviewers and themes that arose during the interview.

### Data management and analysis

All interviews were digitally audio-recorded. Within 24 h of completing each interview, the digital files were downloaded to a secure electronic drive on a Johns Hopkins University server. The files were then sent to a HIPAA-compliant transcription service. The transcripts were proofed against the audio files to ensure completeness and accuracy. After the transcripts were verified, they were analyzed by a team of four coders using Dedoose software (25), using content analysis, an inductive analytical approach to develop themes and meaning from qualitative data (26). Four members of the study team coded the data. Study team members independently reviewed 2–3 transcripts and developed initial codes. The study team then met to refine the codes and create a codebook. To train the coders, two manuscripts were coded by all members of the coding study team and discussed as a group to ensure consistency in coding the data. Thereafter, two team members coded each manuscript, and the interviewers discussed coding disagreements to ensure consensus.

Coders used an iterative process to code data collected from interviews. The first level of coding consisted of *a priori* codes developed from the interview guide domains and *in vivo* codes derived from participants’ transcripts. The coders created analytical memos to capture their impressions about the interviews, the analytic process, and any emerging themes. The second level of coding consisted of pattern coding, where the existing codes were collapsed into similar, smaller categories (26, 27). The categories were based on the similarity and differences among the participants’ responses regarding their incarceration experiences during COVID-19. The codes were then sorted thematically based on substance and content (28).

## Results

### Sample characteristics

We screened and interviewed ten formerly incarcerated individuals released in Maryland during the pandemic. All participants who were screened for the study were eligible for enrollment. Most of the participants were released within the first 6 months of the pandemic. The sample was equally split between men and women, with 60% (*n =* 6) African American and 40% (*n =* 4) White. Seven participants were incarcerated in state or federal prisons and two in jails (one did not report the site of incarceration). Further participant characteristics are in Table 1. Four themes emerged from participants’ descriptions of their experiences: (1) distress from fear, vulnerability and lack of knowledge about COVID-19, and uncertainty about how to protect themselves, (2) few COVID-19 prevention and response protocols and inconsistent implementation, (3) lack of access to programming and communication, and (4) the absence of preparation for release and lack access to usual re-entry services and protection from COVID-19 after release.

**Table 1:** Participants’ self-reported demographics.

Characteristic	*N*
Age in years, mean (range)	43.5 (30–66)
Release date^a^	
April–June 2020	3
July–Sept 2020	3
Nov–Dec 2020	1
Type of facility where incarcerated^a^	
Jail	2
State prison	5
Federal prison	2
Gender	
Cis-Female	5
Cis-Male	5
Race	
African American	6
White	4
Highest education^a^	
10th grade	1
Some college	4
Associate’s degree	1
Bachelor’s degree	1
Graduate degree	1

a Missing data for some participants.

### Distress related to COVID-19

Some participants discussed the early days of the COVID-19 epidemic when the public had little knowledge about COVID-19—what it was, how it was spread, and who was at risk. Participants described receiving initial informal information from T.V. news or the staff at the facilities. As a result of limited information, with some from non-medical sources, the participants had fears related to COVID-19 and how it could affect them. For example:

You know, you figure, a year ago, they were still trying to learn about it [COVID-19]. You know, they… I mean nobody really knew what was going on. You didn't know how it was being transmitted. So, we were on lockdown. (Participant 6)

So, I mean, for me, it was just… it was fearful about not knowing because it was, like I said, it [COVID-19] had just started taking off, and all we could hear about it, really, was with what the correctional officers told us, and then what we would hear on the news far and few in between when we could see the news. So, just the not knowing about it really fully and then being confined was just, yeah, I would say it was very fearful and very stressful. (Participant 10)

While fear precipitated by COVID-19’s unknowns was prevalent across communities, it was exacerbated for incarcerated individuals who, by virtue of their incarceration, had limited means to obtain information. Another participant discussed distress related to contracting COVID-19 and hearing about other residents getting sick and feeling vulnerable.

I think a couple of people died from it. Yeah, a few people died. And, uh, basically, they didn't… Uh, as far as I'm concerned, they didn't do enough – far as cleaning – far as, uh, helping… (Participant 8)

Their distress was laced with critiques of the facility’s shortcomings in infection control. Participants reported distress manifesting as fear, which may have been amplified due to their inability to obtain information and access to preventative measures, like personal protective equipment. Unlike their counterparts in the community, incarcerated persons had to rely on prison administrators and staff and limited access to media to provide pertinent information and education about COVID-19 and to adjust physical spaces to prevent COVID-19 transmission.

### Few COVID-19 prevention and response protocols and inconsistent implementation

Study participants described limited COVID-19 prevention and response protocols and inconsistent enforcement by prison officials. A few participants reported that officials told them of ways to keep the spread of COVID-19 down, which included implementing lockdowns, handing out masks, and telling residents to socially distance themselves; however, protocols were limited and differed substantially by facility.

They told us to wear a mask. They told us to, um, social distance. They told us to wash our hands. They also put up signs, um, re-reiterating those things. And that was basically it. Um, most of the information I think, came from the warden of the jail. (Participant 2)

Nothing got implemented until, like I said, it really got bad, and that was probably three months in, maybe two months in. Then they implemented masks, they gave everybody masks, and that was about it, though. And told everybody, you know, if you don't feel good, put in a sick call, try to practice, you know, staying away from each other… But for the most part, no, there was no type of, uh, protocol or anything in line for it, that's for sure. (Participant 10)

Several participants described receiving personal protective equipment (PPE) and appropriate cleaning supplies only after they or their families advocated for the prison or jail to provide PPE.

…one of the things that we were kind of upset about because we kept pressing them about getting bleach and cleaning supplies and stuff, and it was already a pain in the butt to get them, and we couldn't get them… Like, we got to beg for cleaning supplies. That's… that just seems ridiculous. (Participant 10)

… they started giving us bleach. You know what I mean? They started giving us bleach. I think, after a couple families – people started calling their families, and we'd all have hand sanitizer and this, that, and the third. They got a lot better because, you know, there's nobody wanted to, umm… You know, none of the staff wanted you to call the head office umm, umm, any of that. Umm, so, with that being said, they started, uh, giving us more chemicals to clean and things like that, yeah… (Participant 6)

… we had to try to stay socially distanced… which was impossible because we live in dormitory area, in dormitories… and, uh, that, you know, we've got to keep our places clean, and we had to wear our masks once they start putting the masks out, started giving us masks. But they first started giving us masks. We had to keep them so long, and then we complained… (Participant 7)

Participants also said that some of the rules were inconsistently implemented and described how the strictness or leniency of enforcement of COVID-19-prevention protocols varied among personnel and facilities. This inconsistent enforcement of prison COVID-19 practices was often dependent on who was working that shift.

Then they started giving us the regular surgical masks… Sometimes… it depends on the officers that work the unit… Some officers came and offered you masks. Other officers didn't even care… (Participant 7)

C.O.s do not all abide by the same standards. They all do their job differently, no matter what anyone tells you. Certain correctional officers are sticklers for certain rules and some correctional officers are very friendly and – and make friends with the inmates. So, no matter what anyone says, the standards are not the same. (Participant 9)

Infection control measures were often implemented in circumstances that mimicked solitary confinement.

We were kept in isolation. Our mail was restricted. We couldn't get books or anything that would normally be allowed because of the virus possibly living on, you know, paper. So, isolation is 23 hours a day, no visitors, and our mail were restricted. (Participant 1)

Participants also expressed concern about the limited COVID-19 protocols for carceral staff and felt that the facilities were, “like a revolving door.”

… I was definitely concerned because they were rumors were going around that certain guards were getting it… because… you're very vulnerable in jail and it's, like, you have to come in contact with these guards, C.O.s, correctional officers, deputies. And the deputies go out into the world, and you don't know what they're doing. You don't know how they're interacting with people, if they're going to large-scale functions with a lot of people, and you don't know what type of measures that they're taking to stay safe. And these people, it's like a revolving door, you know, that it's in and out. Every day there's a new set of deputies on shift, and they are the ones who bring the virus into the jail. (Participant 9)

Well, I remember we had, um, maybe like in mid-February, we had a, um, town hall meeting. And it was like the captain of the prison, the warden, and they came, and they were like, um, listen they have this pandemic going on. And, um, we have to keep everyone safe. We're going to be locking the prison down. So, I remember asking the question, because they were like we have to lock you all down for your safety. We have to make sure, you know, that it's not spread that way. I remember asking the question to-to the captain. So, how are you all going to protect us from you? And the… I remember specifically the captain saying to me, ‘don't worry about us.’ (Participant 4)

Incarcerated individuals also expressed a lack of policy for institutional transfer of residents from other carceral facilities which could heighten risk of infection from commonly used items such as telephones, which were perceived as a risk for transmission.

Yeah, but you had people coming in from other institutions coming into the institution. And people that's leaving the institution, they'll be on the same floor at the same time, having to use the same computers, phones, and all that kind of stuff… (Participant 7)

Taken together, responses suggest that COVID-19 protocols differed significantly by carceral facility and were differential enforcement by prison and jail staff.

### Lack of access to programming and communication

Some of the participants described reduced access to services, programming, and activities that ease the burden of incarceration, including access to persons compared to communicating with prior to the pandemic. Such changes were implemented as an infection mitigation strategy. Some participants’ levels of distress related to the pandemic increased because of losing contact with the outside world.

Some participants expressed frustration with newly limited access to medical care during the pandemic. Access to routine healthcare services such as sick calls for acute complaints or chronic disease clinics were interrupted or slowed to make space and resources for COVID-19-specific care, creating a secondary health crisis that exacerbated the health conditions of non-COVID-19 patients with serious health needs. One person stated that, even for serious health issues, they had to wait until their unit was scheduled to go to the medical unit for care. For both COVID-19 and non-COVID-19-related concerns, participants said that they frequently needed to wait to consult with medical staff until their unit was scheduled to go see the medical unit. They also reported a lack of opportunity to inform officers that they needed medical care. Even then, people were not seen for COVID-19 unless they were symptomatic.

The medical people come to the unit, and you tell them what's wrong with you. Then they… they'll send some pills or whatever they think you need, it's cream or whatever, back to the unit. But you weren't really seeing nobody unless you was actually sick from COVID. That went on for a while, almost all of 2020. (Participant 7)

You just had to wait until an officer came around for you to be able to tell them that you didn't feel good. It was much more difficult to get medical attention. (Participant 5)

Participants also noted that facilities switched from in-person visitation with attorneys and their staff to phone or video visits. Participants reported mixed experiences in their access to counsel during the pandemic. Some stated their communication with their lawyers did not change.

My communication with my lawyer did not change. I was able to check when my next hearing was through the kiosk. There was a kiosk system in the block. (Participant 9)

Others felt it was more difficult to access their lawyers, but they still felt adequately represented.

I didn't feel like I received a substantial change in representation. I just felt, you know, I mean, kind of everybody had to go through the motions all the time. So, I felt that I was adequately represented. But it was more difficult. We were able to call, and visits were limited to once per week unless there was a court date that week. And sometimes we did it [via] telecommunication. (Participant 1)

For some participants with families, however, limitations on visitors meant they felt they had to choose between calling their family or their lawyer during their limited phone time. Some were not able to reach out to their legal teams during the small amount of limited free time each day, sometimes limited to 30 min each day. This made it difficult for them to arrange meetings, get information, or to plan for when they were released.

Yes, so they completely shut down all visitation, including attorney visits. And so, it, the only way you could communicate with an attorney would be the email or phone. But there was no physical communication with an attorney. Which, you know, made it very hard for people to communicate with attorneys when I can only come out for 30 minutes a day. I have children. I need to make a phone call. You're only gonna get one phone call in that 30 minutes. Yeah, so it-it-it made it hard for people to, to be able to communicate the way they should have. (Participant 4)

Several participants mentioned that one of the few positive changes they experienced while incarcerated during COVID-19 was free and extended call time.

Yeah, you could make calls. And, uh, the BOP [Bureau of Prisons] did… since the COVID broke out, the phone calls was free. That’s the only real thing they did. They made the phone calls free, and they, uh, raised the, uh, minutes from 300 minutes a month to 500 minutes a month. Basically, that’s the only thing they did during the COVID. (Participant 7)

Several participants reported that recreation and other programming were shut down. The lack of access impacted participants in profound ways, from losing access to substance use disorder recovery programs to religious services and employment. One respondent said, *“*Any kind of extracurricular – any kind of extra outer, I mean, like, privilege type thing was canceled, and you had to deal with it.” (Participant 9) One woman said she lost her ability to help tutor other women in GED classes, which not only represented a limitation of an important prison rehabilitative program but also a loss of purpose.

Yeah. It was just… it was difficult. Because, uh, you know, I was staying sober in there. But there was no recovery program to wrap your hands around. I had a big book. I was able to do some readings. I was able to, you know, pray and do the things that I do to keep myself sober. (Participant 3)

So, what did happen, again, is those programs were all canceled. No programs happened. There were no classes. It was just you were in the block in your cell, you know, in the block on common areas and in your cell for the whole time for the remainder of your stay. There was no church on Sundays, there was no… there was nothing. There was no chaplain, no classes, no financial classes. Every single solitary program was canceled. Haircuts were canceled. Any kind of extracurricular privilege type thing was canceled, and you had to deal with it. (Participant 9)

And the education department was completely closed down. 'Cause I was working there four days a week, teaching, um, other women… h-helping other women acquire their GED. So, to go from at least having some activity in prison to being stuck in our cells, yeah, it was hard. It was really hard. (Participant 3)

This cessation of classes that had brought interaction with the outside world, content that could help prepare people for release, and ways to pass the time further cultivated feelings of distress and isolation. Outdoor recreation time was also limited in many cases. One respondent shared, “… we did not have rec for a while, a long while, and then they would let you outside for supposedly a[n] hour, but we never got a[n] hour.*”* (Participant 7) The culmination of these changes felt especially isolating for participants, who expressed similar thoughts.

… and, uh, during the, the lockdown period, though we, we didn't have rec for a while, a long while, and then they would let you outside for supposedly an hour, but we never got a[n] hour. But if you get out today, you might not get out 'til three, four, five days later again. And then they didn't want you to work out in the unit. It's sad. - PID 133 (Participant 7)

Participants reported reduced access to medical care and legal services, as well as other resources such as Narcotics Anonymous, Alcoholics Anonymous, and church services. Due to the discontinuation of visitation hours and reduced or no alternatives for communication channels, participants additionally reported feeling disconnected from family and friends.

### Disruptions to the release process

Almost all the participants said that COVID-induced changes to the release process impeded their preparation for and reintegration in the community. Some participants were not notified in advance of their release and did not have time to prepare or to take advantage of re-entry programming that may have been available. This created stress and anxiety as several were incarcerated prior to the pandemic and were thus not prepared to navigate with limited resources and support during COVID-19.

The majority saw their release process as abbreviated, which caused a lot of uncertainty concerning how they would survive in the community.

Um, I literally had no days. It was like literally they came to the cell, and they were like, um, so it was more or less hours. It was like you're being immediately released. We're putting travel plans together. And then maybe two hours later I was released. (Participant 4)

The abruptness of the notification for release left participants confused and ill-prepared to return home. One participant described how their post release plans were made invalid by the abbreviated process.

So, my plan was to be able to, um, get into either a halfway house or a transitional program and just leave from the prison and-and go into, um, you know, the program that way. Um, and because I was immediately released, um, I didn't – like obviously, I didn't have that option. And so, I let the staff at the prison know that I didn't have an address. Um, and I really didn't know where I was going to go. Was it any way, I specifically said to them, is it any way you all can contact a program, um, in my area and get me housing, some place to sleep? Um, so I was like, um, and the lady was like, “we can't do that on such short notice, so just make up an address because we have to get you out of here.” (Participant 4)

Another participant described how it was only the kindness of a custody officer that let them to make a last-minute call to their family to assist in the transition process.

Um, and I didn't know that I was coming home when I did. I found out the night before, at about 9:30. Um, and it just so happened that we had a officer who, um, you know, she has a heart. So, she allowed me to get on the phone, um, as – right when she was locking the tier down so I could call my family and let them know that I was being released. Um, you know… had we have had another officer, you know, one… who felt differently, then, my family would not have been able to make arrangements to come get me. (Participant 7)

Such individual acts of generosity could not be relied on for everyone to fill in the gaps created by the expedited release process.

… I just had to rely on family to do everything for me… I mean, I'm not, like, every other person and every other inmate; you know? I have a solid family support system. I was released and I was able to live with my mom. (Participant 9)

One participant who did not have family or other outside support gained help from a probation officer. However, even with this assistance, re-entry was still difficult for the participant.

Well, I definitely did not have personal connections. Um, so I kinda tried to utilize, um, my probation officer as a resource to help me get the thing I need, which was really hard to do because everything was shutdown. Like it took me, it took me almost the entire time I was at this lady's house, which was about four or five months, to get identification. – (Participant 4)

COVID-19 re-entry protocols differed by the facility. A few participants said that individuals leaving prison received COVID-19 tests prior to re-entry into the community. However, it was not consistently implemented, with some residents receiving testing and being quarantined, while others reported being immediately released.

… you were put in, uh, solitary confinement, uh, which is the special housing unit. Um, so, you go in there. So, what they would try to do is… Let's say if you and I were leaving, we'd get out June 21st. They'd put us in the same cell together, no more than two in a cell…for 21 [days]… Give us a COVID test. You get two COVID tests… the basic one. Then they give you the more complex one… and then the reason we need 21 days, because we've got to make sure. They're so backed up that, “We have to make sure that you're, uh, you know, uh, before we release you into the community.” That was a bunch of nonsense, because there were several people who got immediate release from judges, and the judges were like, “You quarantine at home. How about that?” (Participant 6)

Some participants also reported they were not tested for COVID-19 upon release, nor provided with additional information on how to protect themselves from COVID-19 (and by extension friends and family or peers).

No. So I just got released in the street. Nope, did not get tested. Nothing. Like I said, it was just business as usual. They gave me my property and released me right there downtown like… like it was nothing… I didn't… they didn't test me, they didn't tell me go get tested. They didn't do any of that. (Participant 10)

Once released, participants faced multiple structural barriers such as closure of courts, health care clinics, and public benefits offices, and lack of sufficient transportation, which made transitioning back into the community difficult.

Yep. But my… I don't know what I'm - what I'm going to do about it, because my blood pressure medicine is almost ran out. I'm going to see how they handle that. I don't know how they're going to handle that. Yeah, because they only see… When you leave the institution, they only gave - I think they gave me, uh, maybe… I think they might've gave me 60 days' worth. So, I'm on the… I'm working on it. (Participant 8)

So, what I'm basically saying is that this is one of the worst times to be locked up in the pandemic because the court system is shut down and they're like operating on a low level. And all they're doing is postponing the cases. (Participant 2)

It was difficult for some of the participants to navigate re-entry because many organizations and community resources were closed or had limited hours.

Umm, you know, it, it, it just is really… It's really, really scary, just getting out. And, and, and, umm, I guess COVID had everything crazy. Like, no - nothing was open. Like, it, it was – it made the process of, like, getting my I.D. and all kinds of stuff really difficult. Uh, you know, just like you have to apply for Social Security benefits if you qualify for those. You have to apply for food stamps, umm, you know, stuff like that -- just applying for – applying for, for anything that you had to apply for that was a public, uh, thing.” – PID 123 (Participant 5)

For people receiving compassionate release, the experience of release during COVID-19 was often characterized as sudden, with less than 24 h to prepare. Release protocols were not reported to change due to COVID-19 despite the drastically different landscape that released individuals were entering. Services providing housing, social security, identification, and employment were severely impacted by COVID-19, thereby inhibiting returning citizens’ access to these services.

## Discussion

Our study shows that people incarcerated during the height of the COVID-19 pandemic experienced profound distress and fear. In particular, the built-in restrictions on incarcerated people’s abilities to freely access information and control their living space cultivated a sense, often accurate, that prison and jail officials were inconsistently implementing COVID-19 protocols (20, 29). Our study also showed that re-entry planning disappeared and created new challenges to reintegration.

Drawing from experience with other infectious disease outbreaks (e.g., 2009 H1N1), it was widely recognized that people in carceral facilities were uniquely vulnerable to infection (30). Factors like the higher prevalence of certain chronic diseases increased their vulnerability in addition to conditions of confinement that make them more susceptible to infection than the general population. Such conditions include inevitable contact with others in small, often crowded, spaces in which they are confined, lack of access to cleaning and disinfectant supplies, and the perpetual influx and outflux of persons in contact with the pool of infection in the community. In March 2020, the CDC issued its first guidance on the management of COVID-19 for prisons and other detention facilities (31). This guidance used some of the foundations of public health preparedness--prevention, protection, mitigation--to assist facilities in navigating a pandemic within a pandemic. Since then, the guidance has been updated regularly during the course of the pandemic. At the same time, the strategy of lowering the population of these facilities through the release of individuals near the end of their sentences or deemed especially vulnerable to COVID-19 and not a public safety risk began to gain acceptance (13, 32). As a result, many individuals from prisons and jails in Maryland and throughout the country were released to the community under various forms of supervision.

Prison and jail administrators faced serious challenges in preventing the spread of COVID-19 and mitigating its impact. This was due to limited space for social distancing, the absence of or inappropriate spaces for isolating infected individuals from non-infected people, daily movement of staff in and out of the facilities, lack of resources including personal protective equipment and, especially in the early phase of the pandemic, COVID-19 tests. At the same time, the pandemic lockdowns forced community re-entry programs to reduce services. These challenges, moreover, emerged against a background of poor infection control in detention facilities and limited opportunities for incarcerated individuals to be heard on issues regarding their health (33–35).

This study suggests that individuals held in carceral facilities in Maryland experienced concern and distress from COVID-19 mitigation measures, with some perceiving these measures to be haphazard and inconsistent, lacking transparency and fair communication, sometimes with punitive actions, and characterized by a dearth of information. The participants also experienced a lack of control over their ability to protect themselves in the confined spaces of the prisons, which also increased their distress regarding the pandemic. Routine programming and support groups were shut down to slow the spread of COVID-19, as well as non-COVID-19 medical care. This loss of access to programming contributed to increased distresses and a sense of loss, above and beyond what was seen in the absence of the pandemic (36).

Our findings are consistent with studies of COVID-19 response in the federal Bureau of Prisons (21) and in a national study of custody staff, incarcerated people, and medical workers (20). The lack of significant efforts to mitigate the isolation from families and lawyers was common elsewhere as well. A review of state and federal carceral agency policies revealed that 11 states and the federal Bureau of Prisons did not arrange alternative forms of visits with family and counsel, and another six only offered them to some residents (37). A scoping review found that many prison policies to reduce COVID-19 (limited to no visitation, solitary confinement, discontinuing social and supportive activities) increased mental distress and decreased well-being among inmates (38).

Participants also experienced the release process as arbitrary, suffering from a lack of planning, adequate notice, and preparation of individuals for living in the community during a pandemic. They—including some with incarceration experience prior to COVID-19—described re-entry during the pandemic as challenging. Participants felt rushed out with little notice or case management. Upon transition, they found reduced public transportation and limited social services, housing, and other integral community supports for successful reintegration. The process created further uncertainty and stress for them and was exacerbated by the suspension of community-based re-entry programs. The ease or difficulty of transitioning back to the community during COVID-19 largely drew from the relative strength of an individual’s support network, a phenomenon familiar to those who have studied transitioning prior to the pandemic (39, 40). Our findings were similar to those in a study of the release process in federal prisons, including the need to navigate the complexities of seeking and preparing for release without any support, including administrators’ not informing them of eligibility for release or aiding them, or providing them with lists of resources and service providers (22).

The transition process need does not need to be chaotic and stressful as it was for the study participants. In New Jersey, for example, the legislature included provisions on the transition process for individuals released during COVID-19. A study of the release process for individuals with substance use disorders released during COVID-19 there showed that while the program was not problem-free, staff facilitators within prisons who aided the re-entry process, the provision of cell phones, transit assistance, and assistance with continuity of medications, public benefits, and identification documents demonstrated that smooth return in a pandemic is possible (41).

On a broader level, the pandemic both reflected and exacerbated existing power differentials between staff and the people they were incarcerating (20). Both were vulnerable to COVID-19, but the latter were hindered in their ability to gain additional information needed to protect themselves, denied agency, and subjected to practices and rules that seemed arbitrary to participants. Correctional facility administrators’ preparations for a new infectious outbreak must pay heed not only to guidance from the CDC and other expert sources of authority on infection control but must show greater transparency and respect for the dignity and agency of those who are deprived of their liberty. They must be informed about and consulted as carceral routines change and infection control measures are designed and implemented. The study also suggests the need for attention to how the carceral system operates, what its purpose is, and how to avoid converting response to health threats as an element of punishment. While the pandemic posed a unique challenge to administrators, it also represented a canary in a coal mine, as it brought to the surface the arbitrariness of life in a carceral facility, including deprivations of information, voice, agency, and fair treatment that extend far beyond the needs of a system that is designed to house people in a facility they cannot leave. It calls both for reforms that can address unnecessary deprivations and reconsideration of criminal justice laws and policies that result in the incarceration of more people and more people *per capita* than in any other country in the world. While many facilities around the country had disaster plans in place, several had challenges in responding to COVID-19 due to staffing and PPE shortages and quarantine procedures (42). Quality leadership and creative interventions are of prime importance in navigating troubled waters that deeply affect this vulnerable population (43–48).

These participants’ experiences highlight the critical need for new policies that support the health of incarcerated people. For example, it is critical that carceral facilities establish public health procedures and make their plans for health prevention and management publicly available. These preparedness procedures must ensure that incarcerated people can maintain contact with support services (e.g., medical, legal, A.A.) and loved ones when in-person contact is not allowed under public health orders. Additionally, it is imperative that release protocols are standardized across facilities to ensure both individual and community safety.

### Limitations

The small sample size and lack of diverse demographic characteristics, including seven of the 10 participants who attended college and half of the participants identifying as female, are not representative of the population of incarcerated people in Maryland. Additionally, there may be self-selection bias in that those who volunteered to participate in the study may have had stronger critical views of administrators than those who did not; there also could be self-selection bias in that those who, despite the anonymity of the participants in reporting results, were more critical of authorities may have been fearful of possible repercussions against them. Nevertheless, the consistency of responses among participants from different facilities suggests that their accounts are worthy of consideration. Another limitation is that three of the individuals were released very early in the U.S. pandemic, a time when there was uncertainty about effective measures for preventing COVID-19, no guidance had been issued for carceral facilities by the CDC, and those facilities had little experience in controlling infectious disease transmission other than those spread through sexual contact or use of unclean needles.

## Data availability statement

The raw data supporting the conclusions of this article will be made available by the authors, without undue reservation.

## Ethics statement

The studies involving human participants were reviewed and approved by Johns Hopkins Bloomberg School of Public Health Institutional Review Board. The patients/participants provided their written informed consent to participate in this study.

## Author contributions

All authors listed have made a substantial, direct, and intellectual contribution to the work and approved it for publication.

## Funding

This work was supported by Bloomberg American Health Initiative of the Johns Hopkins Bloomberg School of Public Health.

## Conflict of interest

The authors declare that the research was conducted in the absence of any commercial or financial relationships that could be construed as a potential conflict of interest.

## Publisher’s note

All claims expressed in this article are solely those of the authors and do not necessarily represent those of their affiliated organizations, or those of the publisher, the editors and the reviewers. Any product that may be evaluated in this article, or claim that may be made by its manufacturer, is not guaranteed or endorsed by the publisher.
