# Supplementary material for: Pandemic detention: life with COVID-19 behind bars in Maryland
Source: Front Public Health. 2023 Jul 20;11:1217857. doi: 10.3389/fpubh.2023.1217857 (PMC10398335; doi:10.3389/fpubh.2023.1217857)
Supplement: Supplementary file 1 [file Data_Sheet_1.docx]

**Appendix A**. **In-Depth Interview Guide: Released During COVID-19**

Thank you for connecting with us today. We know that coronavirus (COVID-19) has played out differently behind bars than in the community, but we don't know much about how. We'd like to learn from you so that this information can help advocacy groups and other people make sure that incarcerated people are safe.

Today I would like to discuss how COVID-19 influenced your time incarcerated, your release, and how you are currently doing. If you don’t feel comfortable answering certain questions, you do not have to. I would like to start by talking with you about your time while incarcerated.

1. **Can you tell me where you were in custody and how long you had been there when COVID started becoming an issue?**
2. **Can you describe what it was like to be incarcerated, in jail/prison during COVID-19?** [Pause]

PROBES:

- 1. How long were you in custody this time?
  2. How would you describe your health status while incarcerated?
  3. How did you first hear about COVID-19?
  4. What instructions did the [jail/prison] give you about how to protect yourself from COVID-19? {Probe: who, how did they give this info, did they provide guidance on face masks, handwashing, social distancing?)
  5. What did you do to prevent getting COVID-19?
  6. What did correctional officers do to prevent the spread of COVID-19?
     1. Were there rules about the number of people in common spaces (e.g., rec space, pill line) or the dining hall?
     2. Can you tell me about any changes that happened around meal times, housing assignments, cleaning routines, or anything else about the day-to-day in prison/jail?
     3. Did correctional officers wear masks?
     4. What were you instructed to do if you thought you had COVID-19?
  7. Were you ever provided with a face mask or any other personal protective equipment?
     1. Did you wear a face mask? Did other incarcerated people wear masks?

1. **While you were incarcerated, how concerned were you about getting COVID-19? Did your concerns change over time?**
   1. Can you tell me about how other people in the prison/jail were talking about the coronavirus? Were people concerned about getting it?
   2. Did you ever think that you had COVID-19?
      1. Were you tested for COVID-19 at any point while you were in custody? By this we mean a test with a swab. If so, were you ever diagnosed with COVID-19 while in custody?
      2. Were you ever quarantined or isolated? If so, what was this like?
   3. How, if at all, did your communication with your lawyer or legal services change due to COVID-19?
   4. Did you experience any changes in how you could access health care services in jail/prison because of COVID? [Probe – was it easier or harder to access regular health care services?]
   5. Were you involved in any programming, NA/AA or classes while in custody before COVID? [If yes--> Can you tell me how that changed with COVID and how that made you feel?]
   6. Were you getting methadone or buprenorphine for opioid use disorder before COVID?
      1. How did your access to these services change during COVID?
2. Now, I would like to talk about your release. **Can you please describe the circumstances around your release?**

PROBES

- 1. Can you describe things like how and when you learned would be released?
  2. Was your release early or as scheduled?
     1. Do you know if your release was related to COVID-19?
     2. Were you held at a transitional facility?
     3. What obstacles did you face, if any, in getting released? How did you handle them?
  3. Were you tested for COVID-19 prior to release? By this, we mean a test with a swab.
  4. Can you please describe what you were told about COVID-19 when you were released? [probes- need to quarantine? How to prevent the spread? Were you given a facemask or told to wear one?]
  5. Can you tell me about things you were concerned about as you were getting released? [probes- housing? Money? Health care? Infecting my family? Getting prescription medications? Monitoring/parole? Getting public benefits like Medicaid? ]
  6. What kind of help, if any, did you get from either the jail/prison or other groups as you were getting released? [housing, medical care, other re-entry services]
  7. Have you been incarcerated and released before?
     1. If so, how was this time different?

1. I’d like to now discuss how you are currently doing. **What are you doing to prevent getting COVID-19?** [Probe: Wearing a mask outside? Washing hands frequently? Social distancing- keeping at least 6 feet away from other people?

PROBES

- 1. Are you currently concerned about getting COVID-19?
  2. Do you know anyone who has gotten COVID-19?
  3. Where are you currently living? How long have you been there?
  4. If you take any medications, have you been able to get them from a pharmacy?
  5. Can you tell us about any current medical or mental health conditions? Can you talk about what it's like getting health care for your medical conditions or mental health conditions since getting released?
  6. When you are sick and require health care, where do you typically go? How would you pay for it?
  7. Can you tell us about when you have tried to access health services (e.g., doctors visit, mental health services, addiction treatment, including methadone, Suboxone/Subutex/buprenorphine)?
  8. Have you tried to access behavioral or mental health services?
  9. Can you tell me about any ay social services that you have tried to access? Some examples are things like shelters, food pantries, faith-based groups, needle exchange)
     1. Have you had any trouble accessing these services?

1. **Do you have any advice for jails/prisons on dealing with COVID, or advice for people behind bars or getting released during this time of COVID?**
2. **Is there anything that you would like to add about how COVID-19 has affected you or people you are close to, either while you were incarcerated or since your release?**
3. **I would like to end by asking you some basic demographic questions:**
   1. How old are you?
   2. How would you describe your race?
   3. What is the highest level of education that you have completed?
   4. What sex were you assigned at birth?
   5. How do you best describe your current gender identity? (cis-man, cis-women, trans-man, trans-female, non-binary, other)
4. **Finally, we do not currently have a follow-up planned, but if we did, would you be interested in participating in a follow-up study? If so, what is the best way to reach you?**
